# Fabrication of Piezoelectric PVDF/PAR Composites Using a Sheath-Core Fiber Method

**DOI:** 10.3390/polym12102214

**Published:** 2020-09-27

**Authors:** Min Hong Jeon, Yu Rim Lee, Hyeon Soo Lim, Jong Sung Won, Seung Goo Lee

**Affiliations:** Department of Advanced Organic Materials & Textile Engineering, Chungnam National University, Daejeon 34134, Korea; hong831@o.cnu.ac.kr (M.H.J.); rim777@o.cnu.ac.kr (Y.R.L.); hyeonsoo@cnu.ac.kr (H.S.L.); jw2636@cornell.edu (J.S.W.)

**Keywords:** PVDF, piezoelectric, polyarylate, thermoplastic composite, poling, sheath-core fiber

## Abstract

We report the preparation of sheath-core type fibers made from poly(vinylidene fluoride) (PVDF) and polyarylate (PAR) using melt conjugate spinning to fabricate piezolectric composites. The morphology of this sheath-core fiber was determined through scanning electron microscopy. Subsequently, by the compression molding of the PVDF/PAR sheath-core fiber assembly, we fabricated PVDF/PAR composites exhibiting piezoelectric properties. For enhancing the piezoelectric properties, we increased the concentration of PVDF β-crystalline phase in the PVDF/PAR composite through poling post-treatments. The resulting crystal structure of PVDF was confirmed through infrared spectroscopy and X-ray diffraction. A universal testing machine was employed to measure the tensile properties of the PVDF/PAR composites. Finally, through a hydrothermal growing method, ZnO was coated on the composite surface to enhance the piezoelectric properties, which were subsequently optimized by varying the hydrothermal growing conditions.

## 1. Introduction

Poly(vinylidene fluoride) (PVDF) has been the focus of many studies owing to its unique piezoelectric properties [1,2]. Intrinsically, PVDF has four different crystal forms, namely α, β, γ, and δ. Among them, the β-crystalline phase has an all-*trans* conformation with a parallel arrangement of molecular chains, and thus, β-crystalline PVDF has the highest piezoelectricity and ferroelectricity values [3,4,5,6,7,8,9]. In a previous paper, we reported that some post-treatments can increase the concentration of β-crystalline phase in PVDF, thereby improving the piezoelectric properties of the composites made from PVDF [10,11,12,13].

Most piezoelectric studies employ PVDF in a film or sheet type architecture, which restricts the extent of its application [14,15]. However, recently developed PVDF composites show excellent mechanical properties suitable for structural applications such as a skeleton or moving parts of a robot. This has been achieved by a reinforcing procedure that enhances the mechanical and structural properties of the PVDF [16]. We previously reported a liquid crystalline polyarylate (PAR) reinforced PVDF composite fabricated by a sea-island type conjugate fiber method [17]. In that particular composite, PAR and PVDF formed the island and sea component, respectively, and thus, PVDF/PAR composites could be fabricated easily through a simple compression molding technique without any impregnation problems. The resulting composites showed high mechanical strength with reasonable piezoelectric properties.

However, the sea-island type conjugate fiber procedure has several limitations such as a slow fabrication speed, complicated process, high cost, and low production yield. In addition, the conjugation of a polymer with PVDF reduces the piezoelectricity of the composite material Thus, there is a need to find a method for fabricating PVDF composites efficiently, while also enhancing the piezoelectric properties of the composite.

In this study, we report a sheath (PVDF)-core (PAR) type fiber spinning method that is relatively faster and cheaper than the sea-island conjugate procedure. Moreover, by adding an external component, ZnO, we enhanced the piezoelectricity of the PVDF/PAR composites. The PVDF/PAR composites were prepared by the compression molding of the PVDF/PAR sheath-core fibers under different molding conditions. The composites prepared under the optimal condition were post-treated by poling. Finally, we coated the PVDF/PAR composites with ZnO through a surface growing method under various growth conditions. As a consequence of the optimal post-treatment and ZnO coating, the piezoelectric properties of the PVDF/PAR composite were found to be significantly enhanced [18].

## 2. Materials and Methods

### 2.1. Melt Spinning of Sheath-Core Fiber

**Figure 1:**
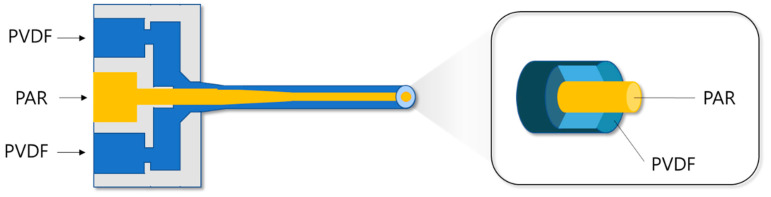
Schematics of the conjugate spinning process for the preparation of PVDF/PAR sheath-core fiber.

The materials used in this study were PVDF (Kynar 70, Arkema, Colombes, France) and PAR (Vecta A950, Celanese, Irving, TX, USA). The PVDF/PAR sheath-core fibers were synthesized by melt conjugate spinning at 270 °C, as shown in Figure 1. The conjugate spinning conditions are listed in Table 1. The sheath-core fiber is composed of a PVDF with piezoelectric properties in the sheath and PAR as a reinforcing component in the core.

### 2.2. Preparation of PVDF/PAR Composites

**Figure 2:**
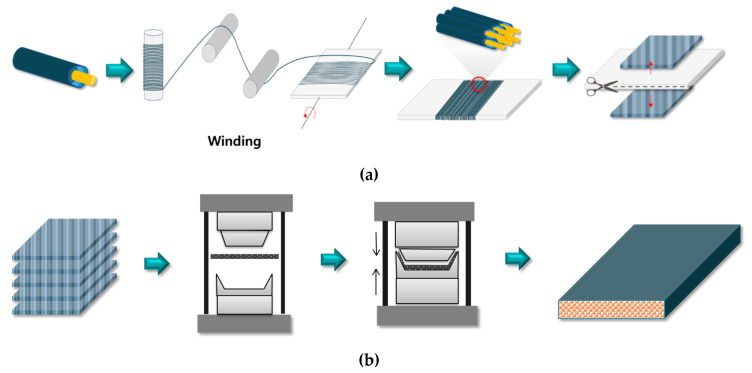
Schematics of the molding process for making the PVDF/PAR Sheath core fiber prepregs (**a**) and composite (**b**).

The PVDF/PAR composites were prepared by compression molding as shown in Figure 2. First, the PVDF/PAR sheath-core fibers were wound, and prepregs were prepared by compression molding at 180 °C and 800 psi for 1 min. Thereafter, a couple of prepregs were laminated to prepare the composite material. The molding temperature (based on the melting temperature of 174 °C for PVDF matrix) was set to 170, 175, 180, and 185 °C, while the molding time and pressure were fixed at 3 min and 1500 psi, respectively.

### 2.3. Post-Treatment of the Composites

**Figure 3:**
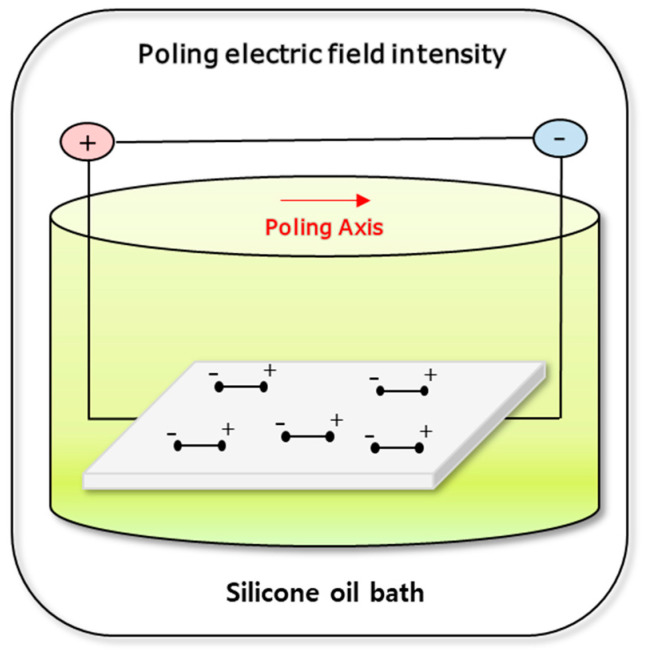
Schematics of PVDF/PAR composite poling process.

To improve the piezoelectric properties of the composite by increasing the relative content of β-crystalline phase in PVDF, poling post-treatment was performed. The poling treatment was carried out using a DC supply (Figure 3). Prior to the poling process, silver paste was applied at both ends of the composite. The poling treatment was carried out for 1–3 h in a silicone oil bath maintained between 110–140 °C (at temperatures above 103 °C, which is the Curie temperature of PVDF), while the poling electric field intensity was set between 200–300 kV/m. Then, it was naturally cooled under DC electric field.

### 2.4. Zinc Oxide Surface Coating

To further enhance the piezoelectricity, ZnO was grown on the surface of the PVDF/PAR composite using the hydrothermal growth method. This method facilitates synthesis using water and heat; the desired material can be obtained by applying heat to the reactant precursors dissolved in water. Thus, the ZnO-coated and uncoated samples will show different piezoelectric properties. This ZnO coating on the composite surface was achieved by initiating seed coating in the ZnO seed solution followed by the growth of ZnO nanowires in the ZnO growth solution. The composite used for ZnO seed coating was cut into a 1 cm × 3 cm piece for the procedure.

**Figure 4:**
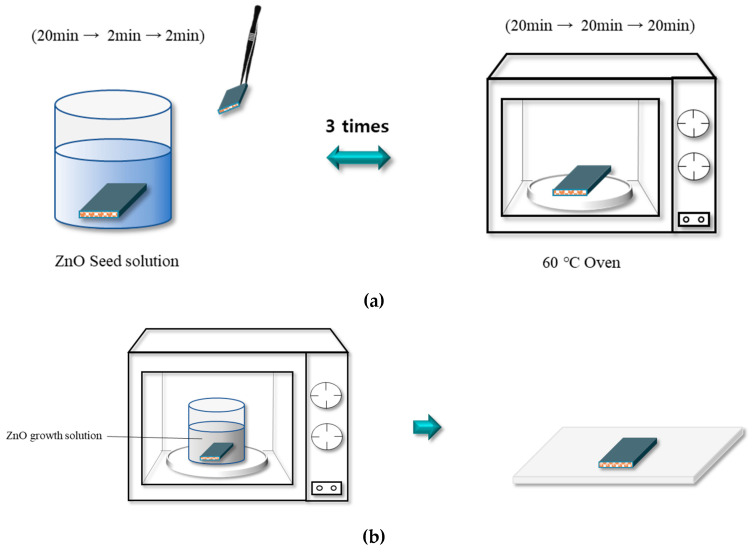
Schematics of the Process of ZnO seed coating (**a**) and ZnO growth (**b**).

The experimental procedure involved stirring a solution of 7.5 mmol zinc acetate dihydrate (8596-4405, Daejung, Siheung-si, Republic of Korea) with 150 mL isopropyl alcohol at 80 °C for 15 min followed by the addition of 7.5 mmol triethylamine (8556-4405, Daejeong) and stirring at 85 °C for 10 min. Thereafter, it was stored at room temperature for 3 h, and then 600 mL isopropyl alcohol was added to the solution to make a 10 mM solution. To coat the composite surface with ZnO, it was first immersed in the diluted ZnO seed solution and then dried in an oven for 20 min at 60 °C. This process was repeated three times; for the first time, the composite was immersed in the seed solution for 20 min, and for the second and third coating, it was only immersed for 2 min (Figure 4a).

This ZnO seed-coated composite, when immersed in a ZnO growth solution and heated in an oven, leads to the growth of ZnO on the surface of the composite. ZnO growth solution can be prepared by mixing 66.7 mmol hexamethylenetetramine (4111-4405, Daejung, Siheung-si, Republic of Korea) with distilled water followed by stirring for 10 min. Then, 40.0 mmol zinc nitrate hexahydrate (8601-4405, Daejung, Siheung-si, Republic of Korea) was added, and the mixture was stirred for 24 h. Table 2 lists the concentration parameters for the solution. ZnO was grown at different temperatures in the range 40–100 °C for 4–8 h (Table 3), which was followed by the washing and drying of the composite (Figure 4b) [19].

### 2.5. Characterization

The morphology of PVDF/PAR sheath-core fiber was examined via scanning electron microscopy (SEM; S-4800, Hitachi, Tokyo, Japan). The tensile strength of the PVDF/PAR composites was measured by a universal testing machine (UTM; Model 4467, Instron, Norwood, MA, USA). Fourier transform infrared spectroscopy (FT-IR; iS50, Nicolet, Seoul, Republic of Korea) and X-ray diffraction (XRD; D/MAX-2200 Ultima/PC, Rigaku, The Woolands, TX, USA) were used to determine the crystal structure of the composite. The piezoelectric properties of PVDF/PAR composites were tested using voltmeters and multimeters (DMM7510, Keithley, Beaverton, OR, USA). The morphology of ZnO growth on the surface of the composite was observed via SEM (SEM; S-4800, Hitachi, Tokyo, Japan), and the crystallinity of the specimen was examined by XRD (XRD; D/MAX-2200 Ultima/PC, Rigaku). The dependence of the piezoelectric properties on the growth temperature, growth time, and concentration of ZnO growth solution was determined using voltmeters and multimeters (DMM7510, Keithley).

## 3. Results

### 3.1. Sheath-Core Fiber Morphologies

**Figure 5:**
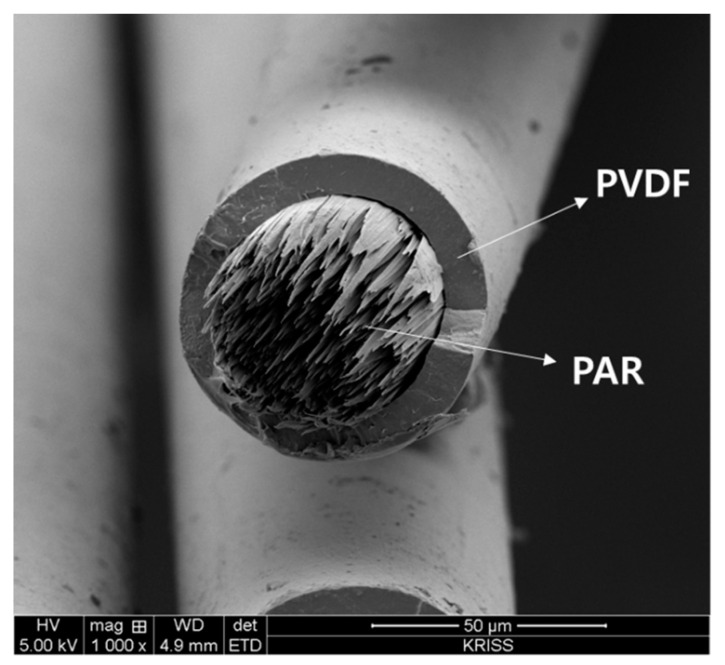
SEM image of the PVDF/PAR Sheath-core fiber.

PVDF/PAR sheath-core fiber was prepared by melt conjugate spinning at 270 °C. In sheath-core fiber, the sheath is made of PVDF with piezoelectric properties, while PAR is used as a reinforcing component for the core. Therefore, the morphology of the fiber is examined in SEM by observing the cross section of the spun fiber. The SEM image showed that in the cross section of the sheath-core fiber, the sheath part was made of PVDF, and the core part was made of PAR fibril structure (Figure 5).

### 3.2. Composite Manufacturing and Mechanical Property (Tensile Properties)

**Figure 6:**
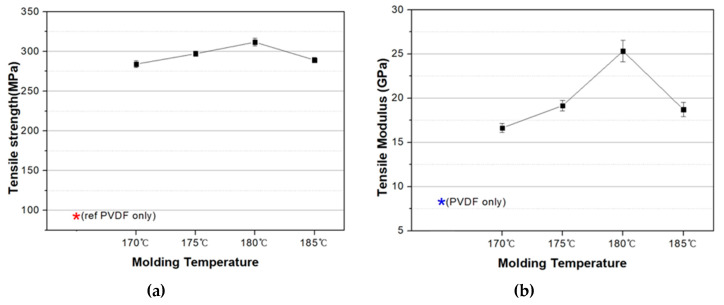
Mechanical property of the PVDF/PAR composites prepared at different molding temperatures: (**a**) Tensile strength, (**b**) Tensile modulus.

The mechanical strength of as-prepared PVDF/PAR composites was evaluated by measuring the tensile properties. We found that the tensile strength and modulus peaked for composites fabricated at 180 °C and decreased progressively for those synthesized below it due to the incomplete melting of the PVDF matrix (Tm: 174 °C). In contrast, at 185 °C, the array of PAR, acting as the reinforcing component, was destroyed, which led to lower tensile strength and modulus than that at 180 °C (Figure 6).

### 3.3. Post-Treatment Effects

#### 3.3.1. FT-IR Analysis

**Figure 7:**
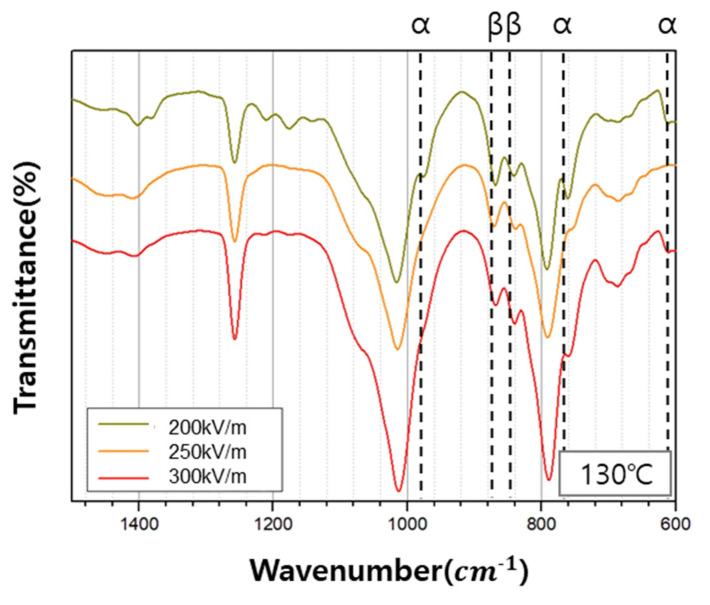
FT-IR spectra of the PVDF/PAR composites measured at different poling electric field intensity.

The changes in the PVDF phase as a result of the post-poling treatment of the PVDF/PAR composite are shown as FT-IR spectra in Figure 7. Here, the bands observed at 976, 765, and 613 cm^−1^ in the FT-IR spectra represent the α-crystalline phase, whereas the bands at 878 and 848 cm^−1^ represent the β-crystalline phase [3,7,20]. It can be easily observed that the contribution of β-crystalline phase increases and that of α-crystalline phase decreases when the polling electric field intensity was set at 250kV/m and 300kV/m. 

**Figure 8:**
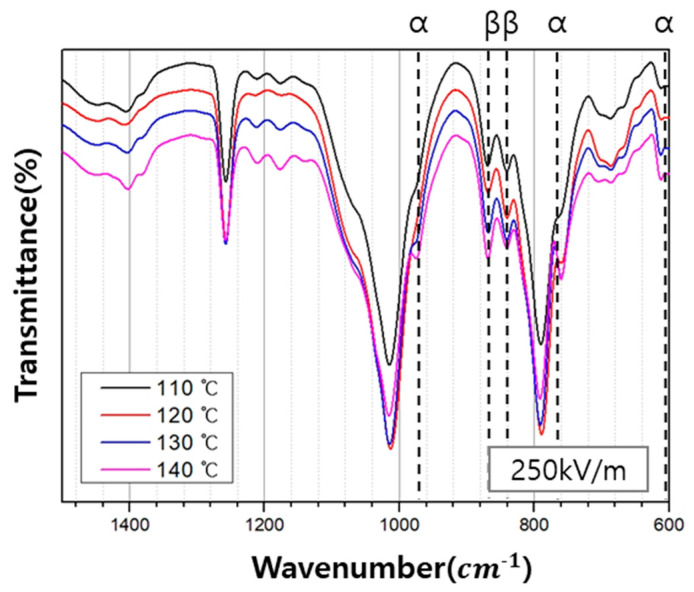
FT-IR spectra of the PVDF/PAR composites measured at different poling temperature.

The extent of β-crystalline phase decreased, and the α-crystalline phase increased when the poling electric field intensity was set at 200 kV/m. Interestingly, for all other poling temperatures, except 110 °C, the β-crystalline phase increased with the corresponding decrease in the α-crystalline phase (Figure 8).

Based on the results of the FT-IR analysis, the relative intensities of the β-crystalline phase were calculated using Equation (1), which is derived from the Beer-Lambert law shown in Equations (2) and (3). Here, $A_{\alpha }$ and $A_{\beta }$ are the absorbances of the α-crystalline phase and the β-crystalline phase, respectively, *L* is the thickness of the specimen, *C* is the average monomer concentration, and $I^{0}$ and I are the intensities of incident and transmitted light, respectively. *K* is the absorption coefficient at each wavenumber, where the values of $K_{\alpha }$ and $K_{\beta }$ are 6.1 × 104 and 7.7 × 104 cm^2^/mol, respectively. *X* represents the degree of crystallinity of each phase, and *F*(β) represents the relative intensity of the β-crystalline phase.

(1) $$F\left(\beta \right)=\frac{X_{\beta }}{X_{\alpha }+X_{\beta }}=\frac{A_{\beta }}{\left(\frac{K_{\beta }}{K_{\alpha }}\right)A_{\alpha }+A_{\beta }}=\frac{A_{\beta }}{1.26A_{\alpha }+A_{\beta }}$$

(2) $$A_{\alpha }=\log\left(\frac{I_{\alpha }^{0}}{I_{\alpha }}\right)=C\cdot K_{\alpha }\cdot X_{\alpha }\cdot L$$

(3) $$A_{\beta }=\log\left(\frac{I_{\beta }^{0}}{I_{\beta }}\right)=C\cdot K_{\beta }\cdot X_{\beta }\cdot L$$

The variation in β-phase content in the PVDF/PAR composite, *F*(β), with the poling electric field intensity and poling temperature is shown in Table 4. As the poling electric field intensity increases, the dipoles are oriented in one direction, increasing the value of *F*(β); therefore, the maximum value of *F*(β), which is 41.3%, is achieved at the highest poling electric field intensity of 300 kV/m. Similarly, as the poling temperature increases, the *F*(β) values increases. It has the maximum value of 45.1% at a poling temperature of 140° C. Therefore, the PVDF β-crystallinity increases with both an increase in poling temperature and poling electric field intensity; however, it depends more strongly on the temperature than the electric field intensity.

#### 3.3.2. X-ray Diffraction (XRD) Analysis

**Figure 9:**
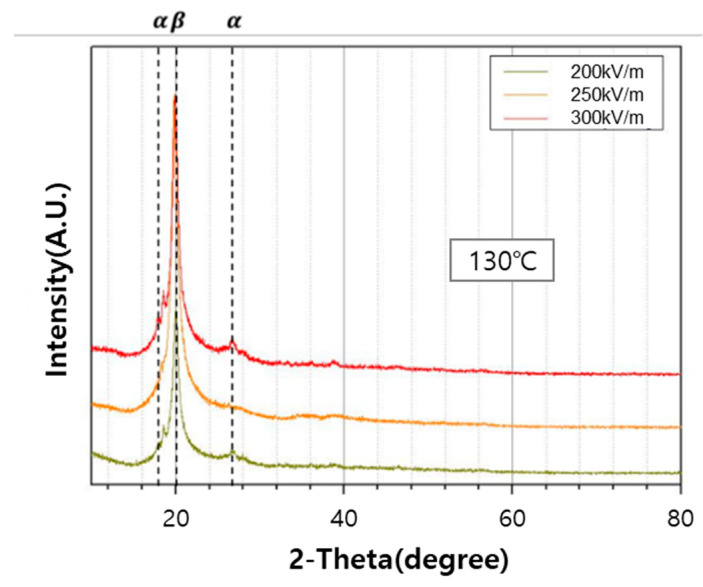
XRD patterns of the PVDF/PAR composite as poling electric field intensity is varied.

**Figure 10:**
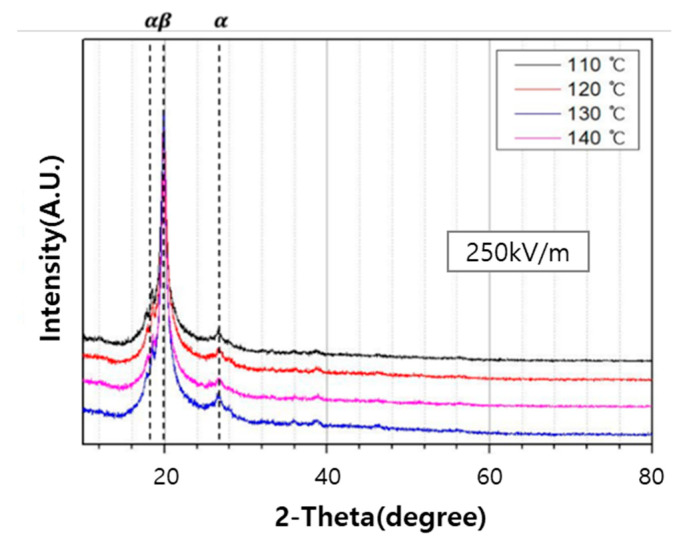
XRD patterns of the PVDF/PAR composite as poling temperature is varied.

Figure 9 and Figure 10 show the XRD patterns of PVDF/PAR composite prepared at different poling conditions. The peaks at the 2θ values of 18.4° and 26.5° can be assigned to the (100) and (020) reflections of the α-phase of PVDF, respectively. In contrast, the peak observed at the 2θ value of 20.3° is assigned to the (110) reflection of the β-crystalline phase [21]. The intensity of the β-phase peak of PVDF/PAR composite poled at 300 kV/m and 140 °C was higher than that under other conditions, which is in excellent correlation with the results of the FT-IR analysis. 

### 3.4. Piezoelectric Properties

**Figure 11:**
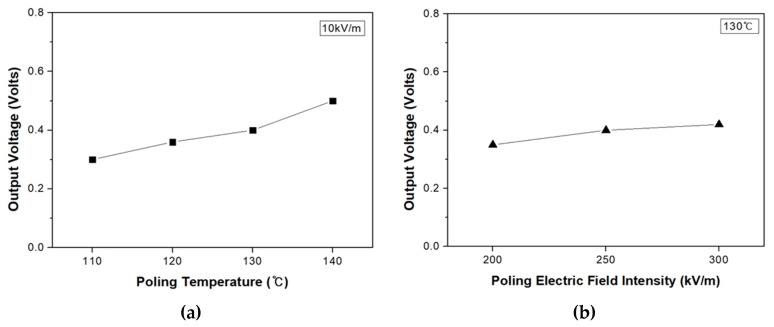
Piezoelectric properties of the PVDF/PAR composite: (**a**) at different poling temperature, (**b**) at different electric field intensity.

The piezoelectric properties of PVDF/PAR composite was measured to post-treatment at various temperatures and poling electric field intensity were measured using voltmeters and multimeters. The composite was prepared by oriented a prepreg made of sheath-core fiber in one direction, and the thickness of the specimen is about 1.2 mm. Piezoelectric property measurement was performed by applying a electric field intensity at both ends in the orientation direction of the composites with a weight corresponding to a pressure of 30 N on the surface of the PVDF/PAR composites. Figure 11 shows the output voltage of PVDF/PAR composites obtained by this method.

The piezoelectric output voltage was the highest when the poling temperature was 140 °C during post-treatment of PVDF/PAR composites, signifying that a higher poling temperature leads to superior piezoelectric properties (Figure 11a). In addition, the piezoelectric voltage was also found to be high when the poling electric field intensity was 250 kV/m or higher (Figure 11b). More importantly, these results indicate that there is a direct correlation between the piezoelectric output voltage and *F*(β) of the PVDF/PAR composite, with the piezoelectric output voltage increasing in an almost linear fashion with an increase in *F*(β) of the PVDF/PAR composite.

### 3.5. ZnO Coating Effects

#### 3.5.1. Morphologies

**Figure 12:**
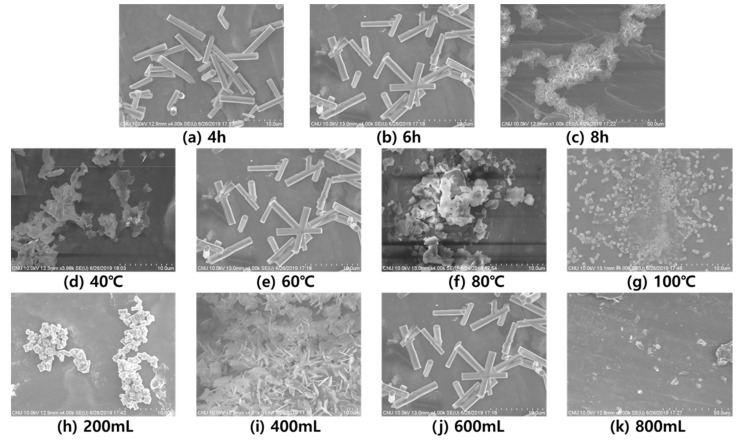
SEM images of the ZnO coated composite surface at different ZnO growth conditions: (**a**–**c**): growth time, (**d**–**g**): growth temperature, and (**h**–**k**): growth solution water. Subfigures (**b**), (**e**) and (**j**) are from one sample at 6 h, 60 °C and 600 mL growth solution water, respectively.

After fabricating the PVDF/PAR composites, ZnO was grown on the surface to further enhance the piezoelectric properties of the composite. Therefore, the SEM analysis was conducted to investigate the morphology of ZnO growth on the surface. The white crystals and the dark areas represent ZnO and PVDF/PAR composites, respectively in the SEM image. The most excellent growth was observed when the ZnO growth time was 6 h. When the growth time was too long, the ZnO particles aggregated. During ZnO growth, the most desirable growth occurred at 60 °C, depending on the temperature, and aggregation occurred when the temperature was high. Moreover, the concentration of DI water in the ZnO growth solution had a considerable influence on the ZnO growth. When the concentration of the ZnO growth solution was too high, severe aggregation was observed, and when the concentration was too low, the ZnO growth did not proceed. The best ZnO growth was observed at a concentration with 600 mL of DI water in the solution (Figure 12). Therefore, the ZnO growth condition greatly influenced the morphology of ZnO growth.

#### 3.5.2. X-ray Diffraction (XRD) Analysis

**Figure 13:**
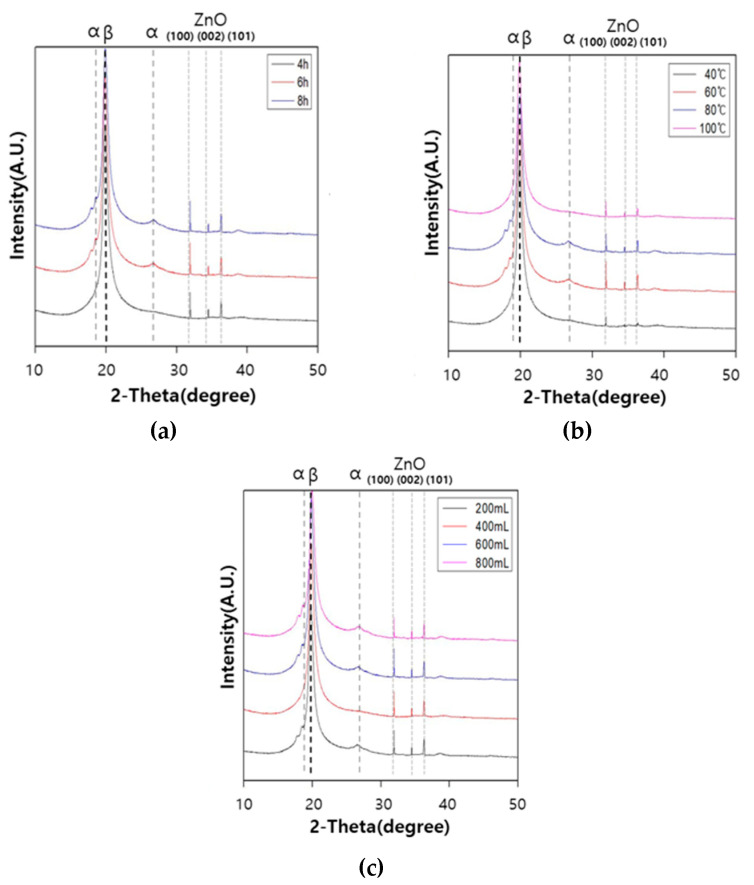
XRD patterns of the ZnO treated composite at different ZnO growth conditions: (**a**) growth time, (**b**) growth temperature, and (**c**) growth solution.

When ZnO treated composites were analyzed by XRD, the diffraction peaks corresponding to the wurtzite crystal structure of ZnO were observed at 31.9° (100), 34.5° (002), and 36.3° (101), as shown in Figure 13. The wurtzite structure is the most thermodynamically stable form for the anisotropic hydrothermal growth of ZnO, owing to a natural tendency to minimize both polar surface and surface energy of ZnO crystal. The wurtzite structure of the ZnO peak was observed in all XRD patterns, irrespective of the ZnO growth parameter [22,23]. Interestingly, except for the growth temperature, there was little or no difference in the peak intensity of diffraction peaks for different growth conditions, indicating that the crystallinity of ZnO is not significantly affected by the other conditions. A variation in the ZnO growth temperature clearly leads to a high crystallinity (high peak intensity) at 60 °C, and low crystallinity denoting insufficient growth at 40 °C. No notable difference in the extent of PVDF α, β-crystalline peaks was observed before and after the hydrothermal growth of ZnO on the composite surface.

#### 3.5.3. Piezoelectric Property

After growing ZnO on a PVDF/PAR composite surface by altering ZnO growth conditions, the piezoelectric properties of the ZnO coated composite were investigated to check for any enhancement/depression. The ZnO growth time did not have a considerable effect on the piezoelectric properties of the ZnO coated composite. Contrarily, the growth temperature of ZnO drastically affected the piezoelectric properties of the composite. 

**Figure 14:**
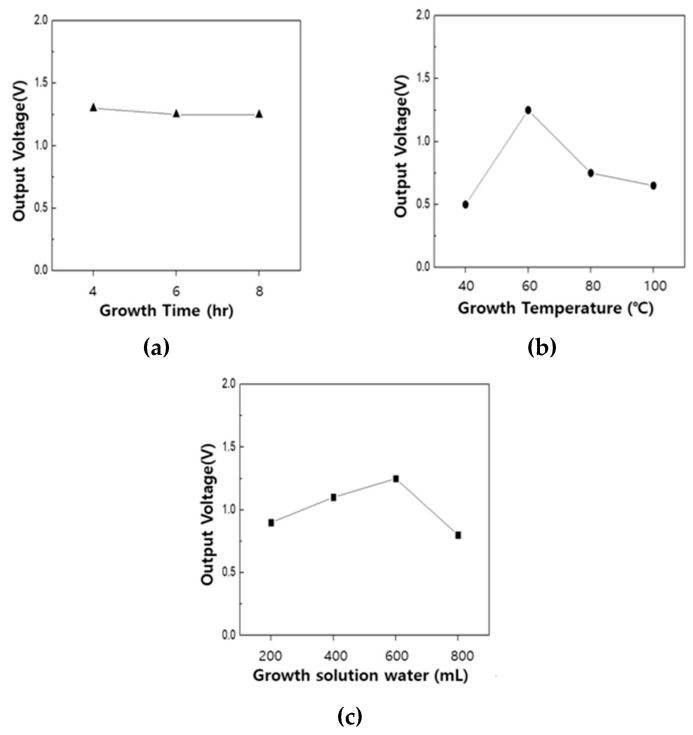
Output voltages of the ZnO coated composites at different ZnO growth conditions: (**a**) growth time, (**b**) growth temperature, and (**c**) growth solution.

The composite synthesized at 60 °C, which also showed the best crystallinity, exhibited considerably superior piezoelectric properties, compared with other growth temperatures. Similarly, the composite treated with 600 mL of ZnO growth solution showed the best piezoelectric properties; however, the dependence of the piezoelectric properties on the concentration of the growth solution was not as strong as that observed for the growth temperature (Figure 14).

Nonetheless, except for the ZnO growth time, the growth conditions employed for ZnO had a great influence on the piezoelectric properties of PVDF/PAR composites coated with ZnO on the surface. The piezoelectric properties of the composites with ZnO grown on the surface were significantly higher than the corresponding PVDF/PAR composites with no ZnO coating on surface. Additionally, compared with the PVDF/PAR composites made from sea-island type fiber in our previous study, the voltage characteristics of the present composite were twice as high.

## 4. Conclusions

In this study, the PVDF/PAR sheath-core fibers were prepared by melt conjugate spinning and later used to synthesize PVDF/PAR composites by compression molding at various molding temperatures. The post-treatment of the composites was carried by poling at different poling electric field intensity and temperature. A molding temperature of 180 °C was identified as the optimal molding temperature for the preparation of PVDF/PAR composites. The *F*(β) of the PVDF/PAR composites increased up to poling temperature of 140 °C because of crystalline phase transition from (α→β). Similarly, during the poling process, *F*(β) increased with an increase in the poling electric field intensity, as the dipoles of the C–F bond of PVDF were aligned along one direction. The highest value of *F*(β) was obtained by applying a 300 kV/m poling electric field intensity to the PVDF/PAR composites. The crystallinity of β-phase of PVDF was more affected by the poling temperature than the poling electric field intensity. Consequently, the best piezoelectric properties of the PVDF/PAR composites were observed for 300 kV/m and 140 °C poling conditions.

The surface of the composite material was then coated with ZnO by employing low-temperature hydrothermal synthesis. Based on the morphology obtained through SEM imaging, we concluded that the best ZnO growth on the composite surface was obtained with a growth time of 6 h, growth temperature of 60 °C, and 600 mL DI water in the ZnO growth solution. The results of the XRD analysis and voltage measurements revealed that the ZnO growth temperature affected the crystallinity and piezoelectric properties much more significantly, compared with the growth time and the concentration of growth solution. The most superior piezoelectric properties among all growth conditions were observed for a ZnO growth temperature of 60 °C. Obviously, the piezoelectric properties of the ZnO-coated composite were two times higher than that of the uncoated composite.

## Figures and Tables

**Table 1:** Spinning conditions for making PVDF/PAR island-in-a-sea fiber.

Sheath component	PVDF (poly(vinylidene fluoride))
Core component	PAR (Polyarylate)
Sheath/Core ratio	1:1
Winding speed	650 m/min
Fiber diameter	52.9 µm
Core component diameter	36 µm

**Table 2:** Preparation of the ZnO growth solution.

Hexamethylenetetramine (mmol)	Zinc Nitrate Hexahydrate (mmol)	DI Water (mL)	Molarity (M)
66.7	40.0	200	0.2427
		400	0.1214
		600	0.0809
		800	0.0607

**Table 3:** ZnO coating conditions.

Temperature (°C)	Time (h)
40	6
60	4
	6
	8
80	6
100	6

**Table 4:** *F*(β) under different poling conditions.

Poling Temperature	Poling Electric Field Intensity (kV/m)	*F*(β) (%)	Poling Electric Field Intensity (kV/m)	Poling Temperature	*F*(β) (%)
130 °C	200	39.2	250	110°C	38
	250	38.8		120°C	41.3
	300	41.3		130°C	42.7
	-	-		140°C	45.1
